# MitoTex (Mitochondria Texture Analysis User Interface): Open-Source Framework for Textural Characterization and Classification of Mitochondrial Structures

**DOI:** 10.3390/ijms27031191

**Published:** 2026-01-24

**Authors:** Amulya Kaianathbhatta, Malak Al Daraawi, Natasha N. Kunchur, Rayhane Mejlaoui, Zoya Versey, Edana Cassol, Leila B. Mostaço-Guidolin

**Affiliations:** 1 Department of Systems and Computer Engineering, Carleton University, Ottawa, ON K1S 5B6, Canada; 2 Tissue Engineering and Applied Materials (TEAM) Hub, Ottawa, ON K1S 5B6, Canada; 3 Department of Health Sciences, Carleton University, Ottawa, ON K1S 5B6, Canada

**Keywords:** mitochondria, microscopy, texture analysis, machine learning

## Abstract

Mitochondria are essential organelles involved in metabolism, energy production, and cell signaling. Assessing mitochondrial morphology is key to tracking cell metabolic activity and function. Quantifying these structural changes may also provide critical insights into disease pathogenesis and therapeutic responses. This work details the development and validation of a novel, quantitative image analysis pipeline for the characterization and classification of dynamic mitochondrial morphologies. Utilizing high-resolution confocal microscopy, the pipeline integrates first-order statistics (FOS) and a comprehensive suite of gray-level texture analyses, including gray level co-occurrence matrix (GLCM), gray level run length matrix (GLRLM), gray level dependence matrix (GLDM), gray level size zone matrix (GLSZM), and neighboring gray tone difference matrix (NGTDM) with machine learning approaches. The method’s efficacy in objectively differentiating key mitochondrial structures—fibers, puncta, and rods—which are critical indicators of cellular metabolic and activation states is demonstrated. Our open-source pipeline provides robust quantitative metrics for characterizing mitochondrial variation.

## 1. Introduction

Mitochondria are highly dynamic organelles that regulate energy metabolism, cellular homeostasis, and signaling [1,2]. Through coordinated cycles of fission and fusion, they remodel continuously between elongated, interconnected fibers and fragmented puncta or rods [3,4]. These morphological transitions mirror changes in metabolic activity and stress responses: elongated networks promote oxidative phosphorylation and survival, while fragmentation is linked to glycolytic shifts, inflammation, and apoptosis [3,5,6]. As such, mitochondrial morphology serves as a powerful proxy for cellular metabolic and functional states [3,6].

Quantifying mitochondrial morphology is therefore critical yet remains challenging. Tools such as the Mitochondrial Network Analysis (MiNA) plugin for ImageJ/Fiji versions 1.0.0 to 2.1.0 have enabled the measurement of network connectivity and branch lengths, but their reliance on skeletonization limits sensitivity when structures are highly fragmented or complex [7]. Subtle variations such as distinguishing rods from donuts or puncta are often misclassified, particularly when image quality or thresholding varies across datasets. Other platforms like CellProfiler or Mytoe provide segmentation flexibility but face similar reproducibility issues, while dynamic approaches such as MitoSPT or Mitometer require live-cell imaging, restricting accessibility for many laboratories [8,9,10,11]. Although deep-learning tools (e.g., MitoSegNet, MitoClass, DNCIC, MitoMo) have improved automation and accuracy, they depend on large, annotated datasets and often function as “black boxes”, hindering interpretability and reproducibility [12,13,14,15].

Beyond morphology, gray-level texture analysis offers an underutilized avenue to capture the subtle spatial organization and intensity patterns that reflect mitochondrial heterogeneity. Texture-based features which have long been used to quantify fibrillar collagen organization to characterize fragmentation, orientation, and local disorder with far greater precision than morphology alone [16,17,18]. Integrating these features provides a pathway to objectively distinguish morphologically similar phenotypes (e.g., rods vs. fibers) and to link image-derived descriptors with functional states such as metabolic activation or oxidative stress.

To address the limitations of existing approaches, we developed MitoTex (Mitochondria Texture Analysis User Interface), an open-source framework that integrates radiomics-derived texture features with supervised machine learning to achieve reproducible, quantitative classification of mitochondrial structures. Unlike tools based purely on skeletonization or segmentation, MitoTex systematically extracts first-order statistics (FOS) and gray-level texture features, gray-level co-occurrence matrix (GLCM), gray-level size zone matrix (GLSZM), gray-level run length matrix (GLRLM), neighboring gray tone difference matrix (NGTDM), and gray-level dependence matrix (GLDM) to capture fragmentation, spatial organization, and structural heterogeneity, followed by recursive feature selection and classifier training. This enables the discrimination of intermediate and morphologically similar mitochondrial phenotypes that conventional tools often overlook.

Finally, we demonstrate the biological relevance of this framework using bone-marrow-derived macrophages (BMDMs), where mitochondrial remodeling accompanies inflammatory activation [19,20,21]. By applying MitoTex to classify mitochondria from untreated and lipopolysaccharide interferon plus interferon gamma (LPS/IFN-γ)—stimulated macrophages, we show that texture-based metrics can sensitively detect activation-related structural shifts, linking quantitative image features to cellular metabolic reprogramming [22,23]. Collectively, this work establishes a robust, interpretable, and accessible computational pipeline that advances mitochondrial characterization beyond morphology. It provides a scalable tool for probing mitochondrial dynamics as biomarkers of cellular state and disease.

## 2. Results

### 2.1. Quantitative Characterization of Mitochondrial Structures

**Figure 1:**
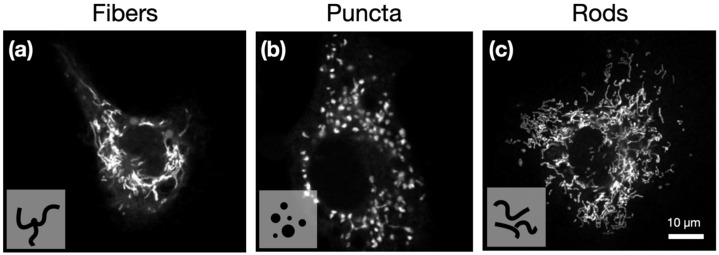
Representative confocal images of single cells, showing mitochondria structures: (**a**) fiber, (**b**) puncta, and (**c**) rods. Images acquired with a 63×/1.4 NA oil immersion objective. All three images were enhanced using ImageJ (Enhance Contrast function, saturated pixels = 0.35%). Mitochondria were stained with MitoTracker deep red (MTDR).

To quantitatively capture mitochondrial diversity, we applied first-order statistics and gray-level texture analysis to confocal images of macrophage mitochondria. This approach enabled the systematic differentiation of fiber, puncta, and rod morphologies shown in Figure 1, revealing textural features that reflect the underlying structural organization and metabolic state of the cells. To see if texture analysis could better capture these features, we first evaluated FOS features, which highlight the differences in the image histogram directly, providing features relating to overall signal intensity, maximum and minimum pixel intensities. As MitoTracker deep red (MTDR) accumulation in mitochondria is dependent on the mitochondrial membrane potential, these analyses only capture active mitochondria. While we cannot exclude the possibility that all aspects of mitochondrial structure were captured in these images, the co-staining of BMDM in MTDR and translocase of outer mitochondrial membrane 20 (TOMM20, conserved outer mitochondrial membrane protein) showed that there was near-complete overlap between these mitochondrial markers (Supplemental Figure S1). Additionally, the gray-level texture analysis applied to the mitochondria images provides novel insights into structural organization, the level of disorder, and fragmentation. FOS and textural features were extracted to quantitatively distinguish between three primary mitochondrial morphologies: fibers, puncta, and rods.

**Figure 2:**
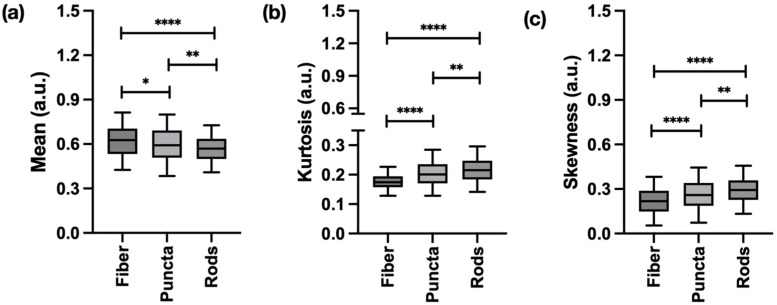
First-order statistic (FOS) features significantly differentiating the three main mitochondrial structures tracked in this work: (**a**) mean, which highlights average signal intensity; (**b**) kurtosis, associated with pixel value distribution/peakedness of the histogram; and (**c**) skewness, which measures the asymmetry of the distribution of pixel intensity. Data represents information obtained from 1334 regions of interests (ROIs) (367 fibers, 546 puncta, and 421 rods). * *p* < 0.05, ** *p* < 0.01, **** *p* < 0.0001.

FOS revealed significant differences in mitochondrial abundance and signal distribution between the three main morphologies evaluated. On average, fibers and puncta exhibited a higher mean signal intensity compared to rods, suggesting a greater overall mitochondrial presence. Kurtosis and skewness were the highest in puncta and rods, reflecting a greater prevalence of intense fluorescent outliers and a larger proportion of low-intensity pixels, which are characteristic of fragmented, distinct structures, as shown in Figure 2.

**Figure 3:**
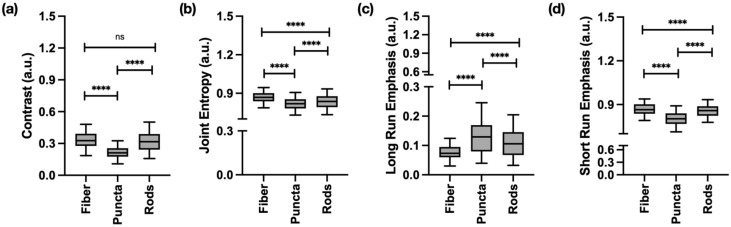
Gray-level co-occurrence matrix (GLCM) and gray-level run length matrix (GLRLM) features significantly differentiating the three main mitochondrial structures tracked in this work. (**a**) GLCM-contrast highlights how well defined the mitochondrial boundaries are. (**b**) GLCM-joint entropy highlights the level of structural disorder. (**c**) GLRLM-long run emphasis produces high values for run lengths that are associated with a long sequence of pixels of the same intensity. (**d**) GLRLM-short run emphasis produces high values for run lengths that are associated with a short sequence of pixels of the same intensity. Data represents information obtained from 1334 ROIs (367 fibers, 546 puncta, and 421 rods). **** *p* < 0.0001; ns: non-significant.

Textural analysis consistently highlighted the unique structural properties of each mitochondrial morphology. Features derived from the GLCM showed that fibers and rods had higher contrast and joint entropy, indicating well-defined boundaries and high structural disorder. In contrast, GLRLM features effectively quantified fragmentation, with puncta showing a higher long run emphasis due to their uniform, homogeneous texture, while fibers exhibited a higher short run emphasis due to rapid changes in pixel intensity, as presented in Figure 3.

Additional analyses using the GLDM, GLSZM, and NGTDM further supported these findings. Fibers and rods were consistently characterized by their textural heterogeneity and complexity, while puncta were defined by their textural uniformity and homogeneity. All textural features obtained can be found in Supplemental Material S1, Table S1.

### 2.2. Results from the Classification of Mitochondrial Structures

To validate the feature-based characterization, decision tree (DT) and one-vs-rest support vector machines (OvR-SVM) classifiers were applied across the full feature set, followed by recursive feature elimination (RFE) to extract the most discriminative features. Classification was evaluated in two contexts: first, to distinguish mitochondrial structure phenotypes, and second, to apply the analysis to untreated and pro-inflammatory conditions in macrophages, highlighting the relevance of this approach in a biologically meaningful model.

**Figure 4:**
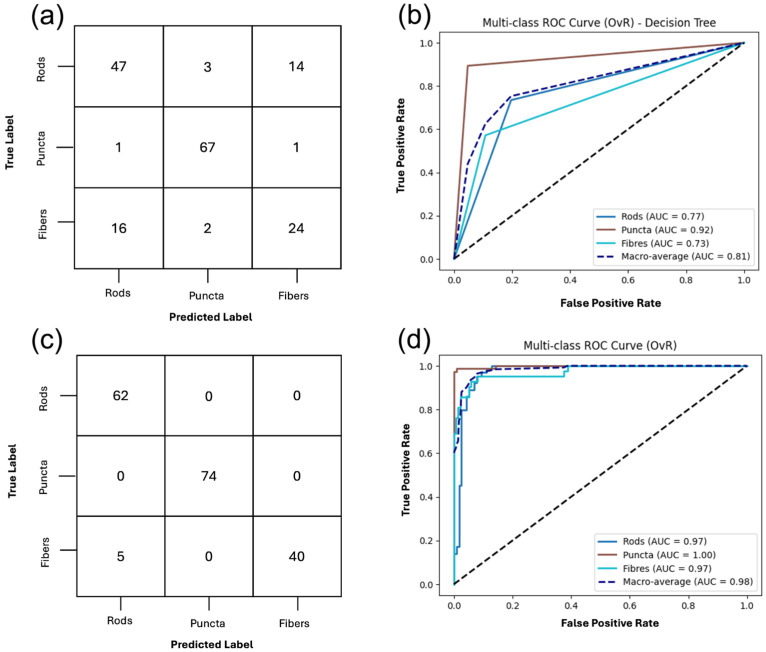
Classification of mitochondrial structures using two supervised classifiers, decision tree (DT) and support vector machine (SVM), on the full gray-level texture feature set. (**a**,**b**) DT classification outputs of puncta, rods, and fibers with a macro-average area under the receiver operating characteristic curve (AUC-ROC) of 0.81 and an overall accuracy of 81%. (**c**,**d**) The one-vs-rest support vector machines (OvR-SVM) classification of the three structures achieves an overall accuracy of 93% and a macro-average AUC-ROC of 0.98. The full classification report is included in Supplemental Material S3, Tables S1 and S2.

The performance of the DT classifier using all extracted features with cross-validation (CV) is summarized in Figure 4, and the full classification report is listed in Supplemental Material S3, Table S1. The model achieved an overall accuracy of 80%. Puncta were classified with the highest fidelity, yielding a precision of 0.91, recall of 0.96, and F1-score of 0.94, indicating that this morphology is highly distinct in feature space. Rods were identified with moderate reliability, with most errors arising from misclassification as fibers. Fibers were the most challenging class for the classifier, with a recall of only 0.55 and an F1-score of 0.60, reflecting substantial overlap with rods; nearly half of true fiber samples were misclassified as rods.

The support vector machines (SVMs) classifier with a radial basis function (RBF) kernel achieved an overall accuracy of 93%, demonstrating strong generalization performance. Puncta were classified with high precision, recall, and F1-score (1.00 across all metrics), confirming that this morphology is highly distinct and consistently separable. Rods were also identified with high accuracy with a precision of 0.93, recall of 1.00, and an F1-score of 0.96, with the few misclassifications arising from fibers being predicted as rods. Fibers showed slightly lower recall of 0.89 but maintained high precision and F1-score of 0.94, indicating that while most fibers were correctly recognized, a small proportion were misclassified as rods as shown in Supplemental Material S3, Table S2 classification report for multiclass SVMs of the full feature set. The receiver operating characteristic (ROC) analysis further supports these findings, with the area under the curve (AUC) values of 0.99 for rods, 1.00 for puncta, and 0.99 for fibers, and a macro-average AUC of 1.00. Collectively, these results highlight the robustness of the SVM classification, particularly in distinguishing puncta and rods, with only limited overlap observed between rods and fibers.

To reduce data dimensionality but most importantly increase interpretability, RFE with CV was employed to identify the top 20 most informative features for classification. These features span FOS, GLCM, GLSZM, and GLRLM; the full list of selected features is outlined in Supplemental Material S1, Table S1.

FOS features such as the 90th percentile intensity, minimum, kurtosis, and energy were identified with RFE. These features reflect global intensity distributions, suggesting that puncta and fibers may differ in brightness distribution and tail heaviness of their intensity histograms.

Several GLCM-derived texture features such as informational measure of correlation (IMC1/2), contrast, correlation, difference average/variance, inverse difference (ID), and inverse difference normalized (IDN/IDM) were selected with RFE, indicating that local spatial relationships between pixel intensities are critical for distinguishing structural morphologies. This suggests that mitochondrial structures differ not only in overall intensity but also in how pixel intensities are distributed relative to one another, reflecting differences in granularity and alignment. These features can highlight the ordered nature of fibers versus the fragmented nature of puncta.

GLSZM and GLRLM features such as zone percentage, size zone non-uniformity, large area low gray-level emphasis, run length non-uniformity, and short-run high gray-level emphasis, highlight the importance of heterogeneity in contiguous regions and run-length patterns. These capture the extent to which mitochondria form continuous elongated structures (fibers), intermediate-sized regions (rods), or small disconnected areas (puncta).

Overall, the selected features confirm the statistical characterization, and the classification of mitochondrial morphologies rely on both global intensity characteristics (FOS) and higher-order texture descriptors (GLCM, GLSZM, GLRLM), with texture heterogeneity emerging as the strongest discriminator between puncta, rods, and fibers. 

**Figure 5:**
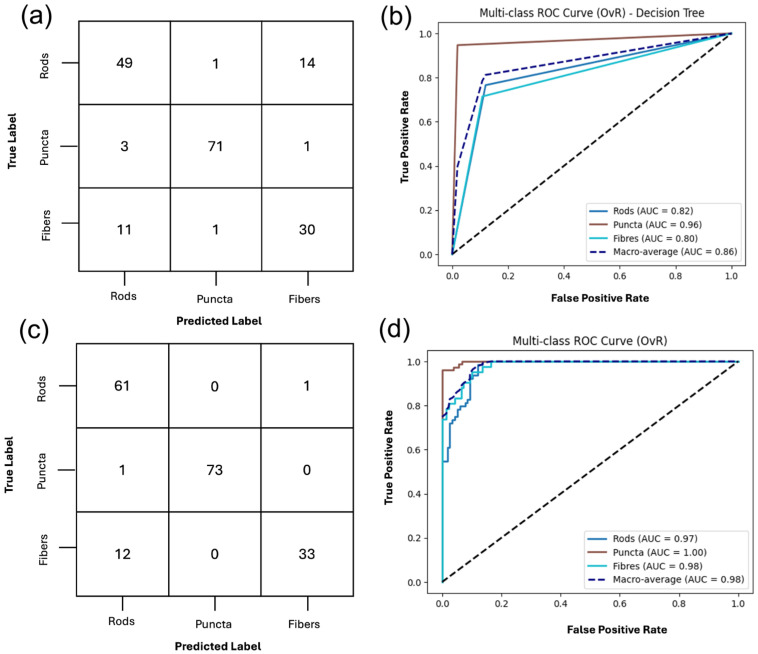
Classification of mitochondrial structures, puncta, rods and fibers, using DT and SVM on RFE-refined gray-level textural feature set. (**a**,**b**) Confusion matrix and ROC curve for DT classification using the twenty recursive feature elimination (RFE)-selected features, with a macro-average AUC-ROC of 0.86 and accuracy of 85%. (**c**,**d**) Confusion matrix and ROC curve for the OvR-SVM, on the same set of twenty selected features, achieving a macro-average AUC ROC of 0.99 and accuracy of 91%. The full classification report is included in Supplemental Material S3, Tables S3 and S4.

The DT and SVM classifiers were retrained using the top 20 features with the aim of improving the classification of mitochondrial puncta, rods and fibers. The classification output of the retrained DT and SVM on the refined feature set can be found in Figure 5.

The DT performed well, achieving an overall accuracy of 85%, illustrated in Figure 5. Puncta were identified with the highest reliability, yielding a precision of 0.97, recall of 0.95, and an F1-score of 0.96, indicating that nearly all puncta were correctly classified. Rods were moderately well recognized, with a precision of 0.78, recall of 0.80, and an F1-score of 0.79. Again, the fibers were more difficult to distinguish with a precision of 0.72, recall of 0.74, and an F1-score of 0.73 reflecting frequent misclassification, particularly as rods. The confusion matrix further illustrates this, as puncta were consistently separated, whereas rods and fibers showed overlap. This suggests that the classifier effectively discriminates the features for puncta but struggles to distinguish tubular morphologies, as shown in the full classification report included in Supplemental Material S3, Table S3.

The top 20 RFE selected features were then used to train the SVM classifier; the model performed well, achieving an overall accuracy of 91%. Puncta were classified with a precision of 0.97, a recall of 0.96, and an F1-score of 0.97, while rods were also accurately identified with a strong recall of 0.92 and F1-score of 0.88. Similarly to the previous classification results, fibers were more variable, with a high precision, but lower recall and an F1-score of 0.86. These results suggest that while the selected features are highly informative for puncta and rods, some degree of overlap in feature remains between fibers and rods. The ROC confirms the trends where the AUC for puncta is 1.00, 0.98 for rods, and 0.99 for fibers, resulting in a macro-averaged AUC of 0.99. These results highlight the discriminative power of the RFE selected feature set, as shown in the full classification report in the Supplemental Material S3, Table S4. 

### 2.3. Classification of Treated vs. Untreated BMDMs

To explore the applicability of this framework in a more biologically relevant context, the same machine learning classifiers were used to discriminate between images taken of control and LPS/IFN-γ-treated BMDM mitochondria. This combination of stimuli induces classical activation of macrophages and pronounced proinflammatory cytokine production [22,23,24,25,26]. This activation state is associated with an increase in mitochondrial fission [22,23,24,25,26].

**Figure 6:**
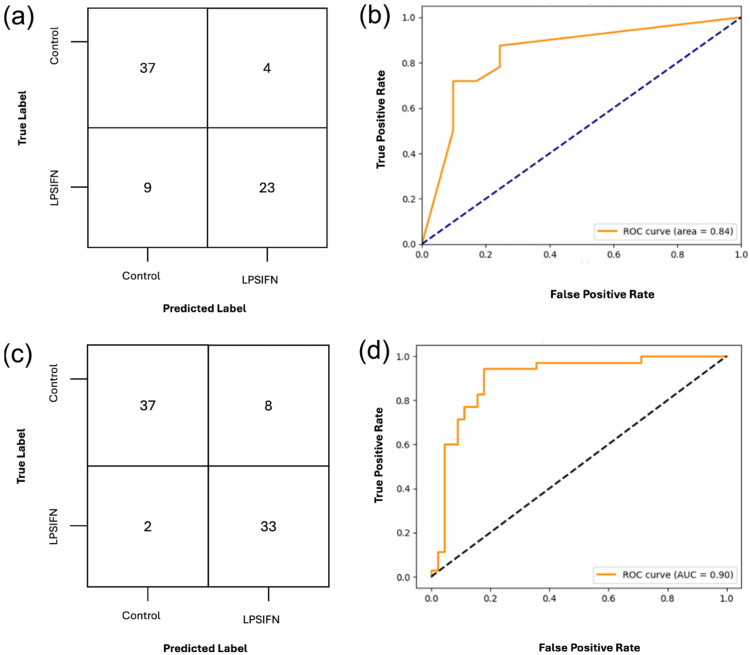
Classification of treated vs. untreated bone-marrow-derived macrophages (BMDMs) using DT and SVM on the top twenty RFE selected feature set to validate and expand our current study. (**a**,**b**) Confusion matrix and ROC curve for DT classification using the twenty RFE-selected features. With an AUC-ROC of 0.84, the DT demonstrates a strong performance. (**c**,**d**) Confusion matrix and ROC curve for OvR-SVM classification using the twenty RFE-selected features. With an AUC-ROC of 0.90, it demonstrates strong generalization performance. The full classification report is included in Supplemental Material S3.

Using the top 20 features selected by RFE, the DT classifier achieved an accuracy of 78% with a precision of 0.78 for the control and 0.77 for LPS/IFN-γ as demonstrated in Figure 6. The recall was higher for control at 0.84 than LPS/IFN-γ treated samples that had a recall of 0.69. The area under the receiver operating characteristic curve (AUC-ROC) is 0.84, indicating a high level of discriminative ability of the model; the full classification report is included in Supplemental Material S3 Table S5.

The SVM outperformed the DT model, achieving an overall accuracy of 88%. The control samples had a precision of 0.95 and recall while the LPS/IFN-γ treated samples had a precision of 0.80 and recall of 0.94. The AUC-ROC was higher in the SVM model, achieving 0.90, indicating that texture features can reliably differentiate between treatment conditions, as illustrated in Figure 6. The full classification report for the SVM is provided in Supplemental Material S3, Table S6.

The top 20 features that were identified by RFE predominantly highlight higher-order texture features derived from the family of gray-level textures. Notably, NGTDM coarseness and complexity were among the highest ranked features, bringing attention to alterations in local intensity variations and structural irregularities. This can be correlated with mitochondrial fragmentation and structural changes due to inflammatory signaling. GLCM IMC1, maximal correlation coefficient (MCC) and correlation reflect spatial dependence and textural uniformity; this could suggest a disruption of coordinated mitochondrial fiber-network organization under inflammatory stress. GLSZM-derived features in particular zone percentage, size zone non-uniformity and zone entropy highlight differences in the distribution of homogeneity. This can be aligned with a shift from elongated fiber-networks to puncta due to the activation of the macrophages. GLDM features-dependency, non-uniformity, and small dependence emphasis capture the altered mitochondrial connectivity under the treated conditions. 

## 3. Discussion

This study presents a quantitative framework for characterizing and classifying mitochondrial structures. The selected FOS and textural features successfully capture key biological characteristics, such as fragmentation, complexity, and structural organization, which are crucial for distinguishing between fibers, puncta, and rods. The results provide a clear quantitative distinction that aligns with qualitative biological observations. Fibers, as fused networks, are quantitatively heterogeneous and low in fragmentation, while puncta, as fragmented structures, are more homogeneous with high fragmentation. Rods, however, occupy an intermediate position, being fragmented but retaining some of the textural disorder of fibers. This quantitative characterization is essential for advancing the collective understanding of mitochondrial dynamics in various biological contexts. Mitochondrial morphology is directly linked to cellular function and pathology. Fused mitochondria (fibers) are vital for processes like quality control and cell survival, while fragmentation is often a hallmark of cellular dysfunction and disease, such as apoptosis or metabolic disorders. For example, fragmented, rod-like structures are associated with metabolic dysfunction and have been linked to increased cell motility and invasion in cancer [27,28].

The semi-automated classification strategy implemented in this study validates the efficacy of the chosen features, providing a valuable tool for biologists to rapidly and objectively quantify mitochondrial morphology. While the classifier performed well in distinguishing puncta, the observed misclassification between fibers and rods highlights the need for a more comprehensive feature set. Future work could add morphological features (e.g., aspect ratio, Feret’s diameter) to improve accuracy. This framework serves as a foundational resource for future investigations into the role of mitochondrial morphology in health and disease.

The use of texture analysis combined with machine learning provided strong classification performance in both discriminating the structures and detecting treatment-induced remodeling in macrophages. In both cases, the SVM outperformed the DT in terms of accuracy, highlighting its ability to capture subtle differences in mitochondrial organization that accompany macrophage activation. This focus on macrophages is particularly relevant, as mitochondrial dynamics are now recognized as central regulators of macrophage immune and metabolic function. During classical (M1-like) activation, mitochondria undergo extensive fission associated with glycolytic reprogramming and pro-inflammatory signaling, whereas elongated networks in alternatively activated (M2-like) macrophages support oxidative metabolism, anti-inflammatory activity, and tissue repair [29,30,31]. By demonstrating that texture-derived features can distinguish these activation-dependent changes, this work establishes a proof of concept for applying MitoTex to investigate immunometabolic remodeling and functional heterogeneity in macrophages. Further studies are required to confirm these findings in other cell types. Given the limitations of using MTDR (dependent on mitochondrial membrane potential), future analyses should also be performed using antibodies against conserved outer mitochondrial membrane proteins (i.e., TOMM20) to ensure we are capturing truly fragmented mitochondria and not active segments along mitochondrial branches [32].

To standardize the workflow, MitoTex integrates feature extraction, selection, and classification into a user-friendly interface, enabling researchers to perform texture-based analyses without requiring advanced programming expertise. This framework supports reproducible and scalable quantification of mitochondrial phenotypes across diverse cell types and experimental conditions.

## 4. Materials and Methods

### 4.1. Cell Source and Sample Preparation

All animal procedures in this study received approval from the Carleton University Animal Care Committee and adhered to the guidelines established by the Canadian Council for Animal Care. Bone-marrow-derived macrophages (BMDMs) were isolated and differentiated using established protocols [24,25,26]. Briefly, bone marrow precursors were isolated from the tibias and femurs of 3-month-old C57BL/BJ mice (Jackson Laboratories) and maintained in high-glucose Dulbecco’s modified eagle medium (DMEM, Life Technologies, Carlsbad, CA, USA) supplemented with 10% (*v*/*v*) fetal bovine serum (FBS), 1% (*v*/*v*) penicillin/streptomycin (PenStrep, Life Technologies, Carlsbad, CA, USA), and 15% L929 fibroblast cell-conditioned medium for 10 days to support differentiation into BMDMs. After differentiation, cells were plated into µ-Slide 8-well dishes (iBidi, Gräfelfing, Germany) at a seeding density of 3×104 cells/cm^2^ in phenol red-free DMEM supplemented with 5 mM glucose, 1 mM sodium pyruvate, 4 mM L-glutamine, 1% PenStrep, 10% FBS, and 15% L929-conditioned media. After resting overnight in a humidified incubator at 37 °C, cells were stimulated for 18 h with 100 ng/ML lipopolysaccharide (LPS, InvivoGen, San Diego, CA, USA) and 20 ng/mL interferon gamma (IFN-γ, R&D Systems, Minneapolis, MN, USA), which is known to induce pro-inflammatory activation states in BMDMs associated with alterations in mitochondrial morphologies (fibers, puncta, and rods).

### 4.2. Cell Staining

Mitochondria were stained using MitoTracker deep red (MTDR, ThermoFisher Scientific, Waltham, MA, USA) and nuclei were counterstained with 4’,6-diamidino-2-phenylindole (DAPI, ThermoFisher Scientific, Waltham, MA, USA). First, 500 nM MTDR in 5 mM glucose DMEM media (without FBS and L929-conditioned media) was added to live cells for 45 min in a humidified incubator at 37 °C. Cells were fixed using 4% PFA (ThermoFisher, Waltham, MA, USA) in phosphate-buffered saline (PBS) for 20 min at room temperature (RT) and subsequently permeabilized with 0.2% (*v*/*v*) Triton X-100 (Fisher Scientific) in PBS for 10 min at RT. Non-specific binding sites were then blocked with 2% (*w*/*v*) bovine serum albumin (BSA; Torcis, Oakville, Canada) in PBS (BSA-PBS) for 30 min at 4 °C. Finally, after washing, the nuclei were stained with 300 nM DAPI in 2% BSA-PBS for 5 min at RT in the dark. Post-staining, the cells were stored in PBS at 4 °C in the dark until imaged.

### 4.3. Imaging Setup

The stained BMDMs were imaged using a Zeiss laser scanning microscope (LSM) 980, a confocal microscope equipped with an Airyscan 2 detector (Carl Zeiss, Oberkochen, Germany). This advanced imaging system is housed at the Tissue Engineering and Applied Materials (TEAM) Hub BioImaging facility (RRID: SCR-022968) at Carleton University. The microscope was configured with specific beam splitters to accommodate the different fluorophores: a 406 nm beam splitter was used for DAPI imaging, and 514/639 nm beam splitters were employed for imaging MTDR/633 dyes. A plan-apochromat ×63/1.4 oil immersion objective (Carl Zeiss, AG, Germany) was utilized to achieve high-resolution images. The imaging parameters were consistently set as follows: MTDR—a digital gain of 680V and laser power of 5%, and DAPI—a digital gain of 700V and laser power of 0.5%, with an LSM scan speed of 5.

### 4.4. Image Pre-Processing

**Figure 7:**
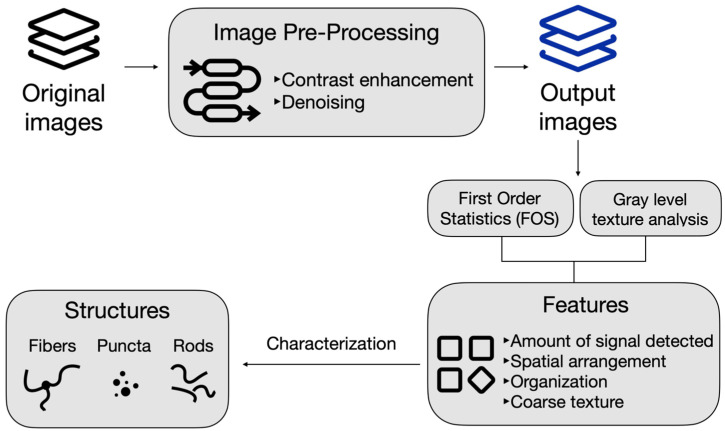
An overview of the image pre-processing pipeline and image feature extraction process to characterize mitochondrial structures.

Prior to conducting the analysis of the structures, an image pre-processing pipeline was established, as shown in Figure 7. The pre-processing steps involved denoising and enhancing its contrast. The image intensity values are adjusted to saturate the bottom and top 1% of the pixel intensities and contrast-limited adaptive histogram equalization was used to enhance the contrast of the output image. This makes it possible to enhance the speckle noise and other forms of noise such as Gaussian noise within the image. The contrast-enhanced image is then filtered using a Wiener filter from MATLAB (version R2023b, MathWorks) and smoothed using a Gaussian filter. The Wiener filter function uses a pixel-wise adaptive low-pass Wiener filter, where the filtration is based on the statistics estimated from a neighborhood of pixel intensities of each pixel [33]. The Gaussian filter smooths the image with a smooth kernel with a standard deviation of 0.5.

### 4.5. Image Feature Extraction: FOS and Textures

After the pre-processing was completed, a region of interest (ROI) size of 69-by-69 pixels was used (pixel size: 0.035-by-0.035 µm), as it was able to encapsulate regions with the same type of structures. The pixel size is smaller than the effective lateral resolution of the Airyscan, since the analysis does not rely on isolated pixel-to-pixel variations; this avoids spatial oversampling. The use of small ROIs was intentional to capture homogeneous regions of mitochondrial structure and avoid bias from background-dominated regions. Since a lot of heterogeneity exists within a single cell, it was crucial to manually select regions of distinct fiber-like, puncta, and rod-like structures. The ROI selection was guided by the mitochondrial morphology described in the literature, particularly by A.J Valente et al., 2017 [7]. Puncta were defined as disconnected, fragmented, and approximately circular structures; fibers as highly interconnected tubular networks; and rods as fragmented tubular structures lacking branching and interconnectivity. The ROIs were manually selected to capture homogeneous regions of a single morphology and were visually inspected to minimize inclusion of mixed or ambiguous structures. A total of three animals were used in this study. In total, 518 images were obtained, and a total of 296 fiber, 482 puncta, and 338 rod ROIs were analyzed, for a total of 1116 ROIs. Once the ROIs were organized categorically, the PyRadiomics library was used to extract image features [34]. A total of 93 features were extracted from each image. There were 5 texture groups that were used in this study, gray-level co-occurrence matrix (GLCM), gray-level size zone matrix (GLSZM), gray-level run length matrix (GLRLM), neighboring gray tone difference matrix (NGTDM), and gray-level dependence matrix (GLDM) [18,35,36,37,38]. The steps taken prior to feature extraction and the description of all features are available in Supplemental Material S1, Table S1.

### 4.6. MitoTex: Mitochondria Texture Analysis User Interface

MitoTex is a Python-based graphical user interface (GUI) designed to extend mitochondrial image analysis beyond morphology-based tools. The interface integrates radiomics-based texture feature extraction, statistical feature selection, and supervised classification into a single workflow. Input images, in tagged image file format (.tiff), are pre-processed prior to analysis. Texture features are extracted using the PyRadiomics library from multiple feature classes, including GLCM, GLRLM, GLSZM, and NGTDM. These descriptors capture mitochondrial heterogeneity, fragmentation, and spatial organization that are not detectable through morphology alone. To reduce data dimensionality and retain the most informative parameters, recursive feature elimination (RFE) was used for feature selection [39]. The selected features were subsequently used to train and evaluate supervised machine learning classifiers, specifically multi-class one-vs-rest support vector machines (OvR-SVM) and decision tree (DT), for phenotype classification across experimental conditions [40,41,42,43]. MitoTex outputs include the following: (i) a feature table (.csv) of extracted radiomics descriptors, (ii) the subset of selected features following RFE, and (iii) classification results, including accuracy, precision, recall, F1-score, and confusion matrices. The GUI was designed to be accessible for both technical and non-technical users, providing an interactive interface for feature selection, classifier choice, and visualization of results. The full code and an executable version are available at https://github.com/Tissue-Engineering-BioImaging-Lab (accessed on 1 January 2026), with a detailed user guide provided in the project README.

### 4.7. Classification of Mitochondrial Structures

To mitigate the risk of overfitting, the number of features used for classification was reduced through a wrapper-type feature selection method, specifically RFE. RFE iteratively trains a model and removes the least important features at each step, ultimately retaining the top 20 features most relevant for distinguishing the three mitochondrial structures. By ranking features based on their predictive importance, RFE ensures that those contributing the most to class separation are prioritized. The choice to retain the top 20 features is based on finding a balance between model performance, overfitting reduction, and interpretability. While the primary analysis focused on the top 20 features, classifier performance was evaluated using repeated stratified cross-validation and bootstrapping to ensure robustness and generalizability of the models trained on this refined feature set. 

A DT classifier was first implemented to categorize the mitochondrial structures using the refined subset of features. A DT classifies observations through a hierarchical branching process, beginning at a root node and applying threshold-based decisions at each subsequent node. Data are directed along left or right branches depending on whether feature values fall below or above a defined threshold. The scikit-learn Python library default decision threshold of 0.5 was applied, where conditional probabilities greater than 0.5 predicted the positive class. 

To benchmark performance, an OvR-SVM classifier was trained in parallel on the same feature set. In this framework, a binary support vector machine (SVM) classifier is trained for each class against all others, and final predictions are assigned according to the classifier with the highest decision function output. The SVMs employed a linear kernel to maximize the margin between classes, thereby enhancing separation in the high-dimensional feature space. 

Model development and training of DT and OvR-SVM were implemented in scikit-learn. For both classifiers, performance was assessed using repeated stratified k-fold cross-validation to obtain stable estimates of accuracy while preserving class balance across folds. To evaluate generalization performance, cross-validation (CV) strategies were tailored to each model type. A 10-fold stratified CV is applied to the DT to stabilize performance estimates given its higher variance. For the OvR-SVM, a repeated 2-fold CV (3 repetitions) is leveraged to balance computational efficiency with variance estimation, as training SVMs with radial basis function (RBF) kernels is more computationally intensive. Stratification ensured that class distributions were preserved across folds, and performance was reported as mean ± standard deviation of accuracy across repetitions. To complement CV, test set predictions were further evaluated using receiver operating characteristic (ROC) curves, with 95% confidence intervals estimated from 1000 bootstrap resamples to account for variability in the estimates [44,45]. This combination of CV and bootstrapping provides confidence in model performance, generalizability, and stability. The detailed steps outlining the DT and OvR-SVM classifier implementations are provided in Supplemental Material S2. All feature refinement and classification models were implemented using Scikit Learn packages in Python 3.11.4 [44,45].

### 4.8. Statistical Analysis

For each of the extracted textural features, an outlier detection and removal process was implemented using the median absolute deviation (MAD) method [46]. Data points that fell outside the defined bounds, determined by incorporating a scaling factor of k = 2.1, were identified as outliers and subsequently replaced with not a number (NaN) values. This scaling factor was experimentally optimized to ensure a sensitive yet strict approach to outlier removal, ensuring that the detection mechanism was highly responsive to deviations and effectively defined the boundaries for outlier identification. The data bounds were established by calculating the median of the dataset, with any value outside the range of the median ± (scaling factor × MAD) being considered an outlier. The MAD calculation, which assesses the variability of data spread, was performed using MATLAB (version R2023b, MathWorks). Following outlier removal, the data underwent normality testing using the Shapiro-Wilks method. Based on the normality test results, two types of statistical analyses were employed for comparisons, both implemented in MATLAB (version R2023b, MathWorks): the parametric Welch’s test and the non-parametric Mann–Whitney U test. These tests were selected to account for the unequal variances observed across different sample sizes. A *p*-value lower than 0.05 was statistically significant. 

## 5. Conclusions

This study established an objective framework for quantitative characterization and classification of mitochondrial and collagen structures from microscopy images. By employing a two-stage approach that combined FOS and gray-level texture analysis, the classifiers were able to effectively differentiate between distinct mitochondrial morphologies (fibers, puncta, and rods) as well as identify potential texture-based biomarkers to identify differences in macrophage activation in a pro-inflammatory environment. The consistent and strong performance of the SVM classifiers, evidenced by high AUC-ROC values for classifications of both mitochondrial structures and macrophage metabolic activity in a pro-inflammatory environment demonstrate the efficacy of using these features for studying metabolic activity. The results provide a quantitative understanding of structural alterations in biological systems. While this work provides a valuable framework, its findings also highlight clear avenues for improvement. Future research should focus on expanding image datasets, particularly by increasing the number of animals to better account for individual biological variability. Incorporating a broader range of features, such as morphological metrics and more robust texture analysis techniques like Gabor filters, could enhance classifier accuracy, especially in distinguishing between morphologically similar structures like fibers and rods. Additionally, in 2D projections, mitochondrial apparent length and branching can be influenced by cell flattening or orientation. Thus, in the feature-space used in this study, there is a close relation between fibers and rods compared to puncta. Future work should incorporate 3D projections to study branching compared to the cell body. Future studies should opt to use a stain that does not inhibit mitochondrial respiration of macrophages, as MTDR inhibits mitochondrial oxygen consumption [47]. Ultimately, a more comprehensive, multi-modal approach combining these features and utilizing more advanced machine learning models holds significant promise for creating a powerful, clinically relevant tool to track changes in mitochondrial structures in the context of disease, aging, and treatment.

## Data Availability

The original contributions presented in this study are included in the article/Supplementary Material. Further inquiries can be directed to the corresponding author.

## Supplementary Materials

The following supporting information can be downloaded at https://www.mdpi.com/article/10.3390/ijms27031191/s1.
