# Supplementary material for: MitoTex (Mitochondria Texture Analysis User Interface): Open-Source Framework for Textural Characterization and Classification of Mitochondrial Structures
Source: Int J Mol Sci. 2026 Jan 24;27(3):1191. doi: 10.3390/ijms27031191 (PMC12897202; doi:10.3390/ijms27031191)
Supplement: Supplementary file 1 [file ijms-27-01191-s001.zip › Supplemental_figure_S1.pdf]

Supplemental Figure S1 – MTDR and TOMM20 mitochondrial overlap

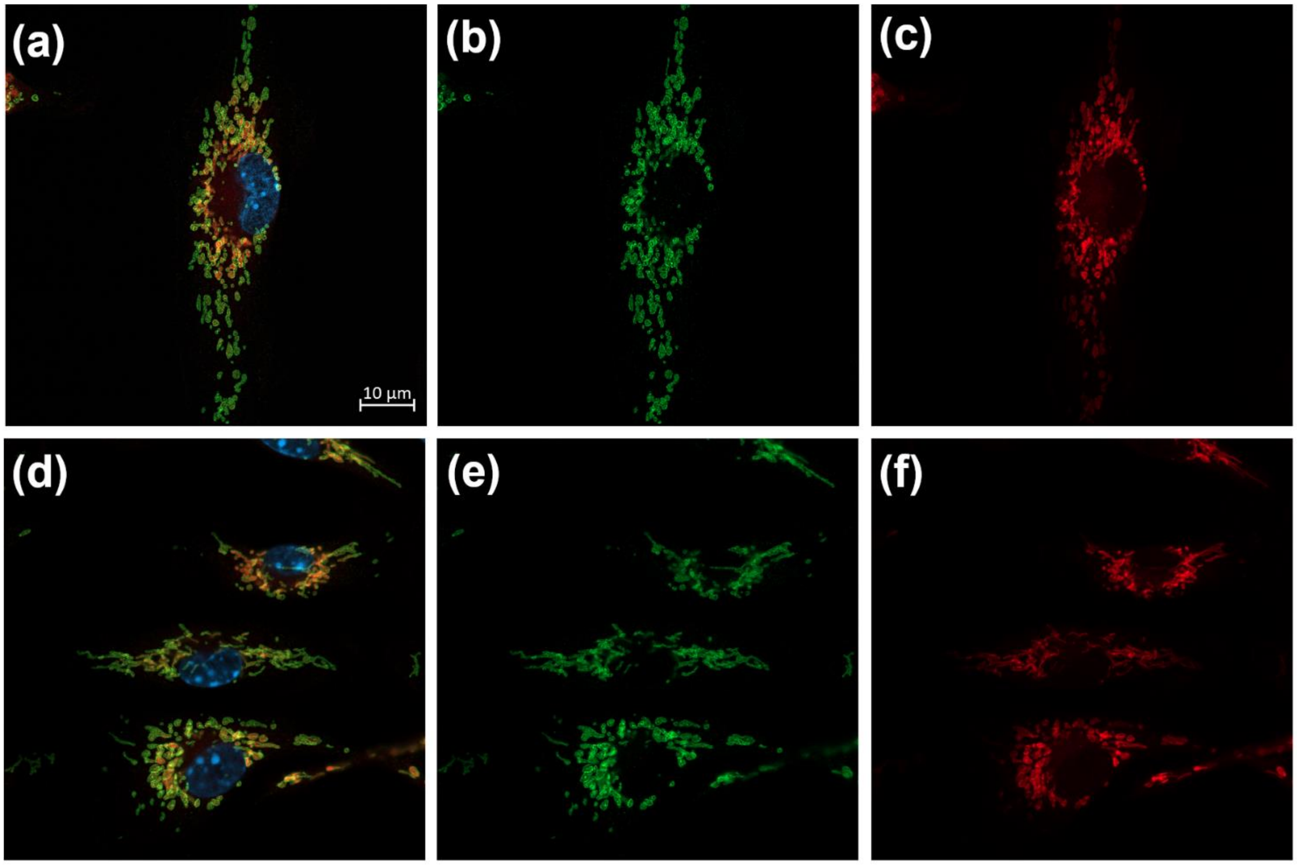

Figure F1. Representative confocal images of cells showing overlap with MitoTracker deep red (MTDR, red) and translocase of outer mitochondrial membrane 20 (TOMM20, green): a) Control single cell bone marrow derived macrophages (BMDM) co-stained with MTDR and TOMM20 showing overlap. Showing high level of fragmentation, b) Control BMDM showing only TOMM20, c) Control BMDM showing only MTDR, d) Control multi-cell co-stained with MTDR and TOMM20 showing overlap. Showing fragmented and interconnected structures, e) Control multi-cell image showing only TOMM20, and f) Control multi-cell image showing only MTDR. Images acquired with a 63x/ 1.4 NA oil immersion objective.
