# Supplementary material for: MitoTex (Mitochondria Texture Analysis User Interface): Open-Source Framework for Textural Characterization and Classification of Mitochondrial Structures
Source: Int J Mol Sci. 2026 Jan 24;27(3):1191. doi: 10.3390/ijms27031191 (PMC12897202; doi:10.3390/ijms27031191)
Supplement: Supplementary file 1 [file ijms-27-01191-s001.zip › Supplemental_material_S1.pdf]

Supplemental Material S1 - Image Analysis and Preprocessing

Prior to feature extraction, a systematic image pre-processing pipeline was implemented to reduce noise, enhance contrast, and ensure consistency across all images. The workflow was designed to optimize texture feature extraction by improving the signal-to-noise ratio and highlighting structural details of interest.

Pre-processing Steps (MATLAB implementation)

1. **Grayscale conversion:** Raw .tiff microscopy images were first converted to grayscale for analysis based on pixel intensities.
2. **Intensity adjustment:** Pixel intensities were normalized using the imadjust() function, which saturates the bottom and top 1% of pixel intensities to improve dynamic range.
3. **Contrast enhancement:** Adaptive histogram equalization (adapthisteq()) was applied to further improve contrast across local regions, making subtle structures more visible.
4. **Noise reduction:** Speckle and Gaussian noise were suppressed using a Wiener filter (wiener2()), which adaptively applies a low-pass filter based on local pixel statistics.
5. **Smoothing:** Final refinement was achieved using a Gaussian filter (imgaussfilt()), which smooths intensity variations while preserving edges.

The processed images were automatically saved in a dedicated output directory with filenames labeled automatically.

Feature Extraction (PyRadiomics)

After preprocessing, regions of interest (ROIs) were selected and categorized into fiber-like, puncta, and rod-like structures. PyRadiomics was then used to extract quantitative features across multiple texture classes.

1. **ROI preparation:**
    - Pre-processed .tiff images were converted to numpy arrays and then to SimpleITK format.
    - A binary mask covering the full ROI was generated to ensure consistent analysis.
2. **Extraction settings:**
    - **Bin width:** 25
    - **Resampled pixel spacing:** None (images were acquired with the same protocol).
    - **Interpolator:** B-spline (sitk.sitkBSpline)
3. **Enabled feature classes:**

- First-order statistics (FOS)
- Gray Level Co-occurrence Matrix (GLCM)
- Gray Level Run Length Matrix (GLRLM)
- Gray Level Size Zone Matrix (GLSZM)
- Neighboring Gray Tone Difference Matrix (NGTDM)
- Gray Level Dependence Matrix (GLDM)

4. **Directional averaging:**
   For features dependent on direction (e.g., GLCM, GLRLM), calculations were performed at 0°, 45°, 90°, and 135°, and then averaged to minimize directional bias.
5. **Normalization:**
   To ensure comparability, extracted features were normalized between 0 and 1, with the maximum value within each structural group set as the normalizer.

All extracted features were exported to .csv and .xlsx formats, with separate sheets for each feature class (FOS, GLCM, GLRLM, GLSZM, NGTDM, GLDM).

Supplemental S1 Table S1- Description of Textural Features

Texture Family	Features	Definition	Biological Relevance
First Order Statistics (FOS)	10/90 Percentile	Intensity value below which 10% (or 90%) of voxel values fall	Captures distribution limits of mitochondrial signal intensity
	Energy	Sum of squared intensity values	Reflects overall fluorescence strength. Where a higher value corresponds to a greater amount of mitochondrial content
	Entropy	Measure of randomness in intensity distribution	Higher values indicate more heterogeneous mitochondrial signal
	Interquartile Range	Difference between the 75th and 25th percentiles. Captures the spread of the central 50% of intensity values	Captures the spread of the mitochondrial signal at the center 50% of the histogram
	Kurtosis	Peakedness of the image histogram	Identifies extremes in mitochondria fluorescence. Where

			sharp peaks suggest clustered intensities
	Maximum	Highest intensity in a given region of interest	Maximum detected mitochondrial signal
	Mean Absolute Deviation	Average deviation from the mean pixel intensity	Quantifies the deviation of mitochondrial signal from the mean intensity
	Mean	Average pixel intensity	Reflects average mitochondrial fluorescence signal
	Median	Middle intensity value	Represents central tendency of mitochondrial signal
	Minimum	Lowest intensity in each region of interest	Minimum detected mitochondrial signal
	Range	Difference between maximum and minimum intensity	Captures overall spread of mitochondrial fluorescence
	Robust Mean Absolute Deviation	Mean deviation calculated within the 10th and 90th percentile	Measure of variability in fluorescence without outliers
	Root Mean Squared	Square root of mean squared intensities	Highlights strong signals
	Skewness	Asymmetry of the histogram	Identifies imbalance between high and low mitochondrial signal
	Standard Deviation	Spread of intensities around the mean	Quantifies variability of mitochondrial signal intensity
	Total Energy	Energy scaled by voxel volume	Reflects total fluorescence signal within the region of interest
	Uniformity	Sum of squared normalized intensities	Where higher values reflect more homogeneous mitochondrial signal distribution
	Variance	Statistical measure of variance of intensity	A measure of heterogeneity of signal
Gray Level Co-Occurrence Matrix (GLCM)	Autocorrelation	Frequency of similar pixel intensities found as pairs	Captures organizational consistency of the mitochondria
	Cluster Prominence	Degree of asymmetry or peakedness in the distribution	Where higher values reflect more irregular, asymmetric textures

	Cluster Shade	Measure of skewness and uniformity of intensity groups	Higher values correspond to greater levels of asymmetry in organization
	Cluster Tendency	The tendency of pixels to form clusters with similar intensities	Captures grouping of pixels with comparable gray level intensities
	Contrast	Difference between neighboring gray levels	Reflect clarity of mitochondrial structure boundaries
	Correlation	Relationship between neighboring intensity values	Captures alignment and structural organization
	Difference Average	Difference between similar and dissimilar pixel pairs	Highlights intensity differences within local regions
	Difference Entropy	Randomness of local intensity differences	Higher values denote disordered architecture
	Difference Variance	Variability in intensity differences	Indicates structural heterogeneity
	Inverse Difference (ID) (Homogeneity) and Inverse Difference Moment Normalized	Local uniformity of pixel intensities	Higher values reflect smooth, homogeneous structures
	Informational Measure of Correlation (IMC) 1 and 2	Measure of complexity	Quantify overall textural complexity
	Inverse Variance	Measure of local homogeneity	High values mean uniform structures
	Joint Average	Average intensity of neighboring pairs	Reflects mean gray level distribution
	Joint Energy (Angular Second Moment)	Uniformity of pairwise intensities	Higher values correspond to more ordered and homogeneous patterns
	Joint Entropy	Randomness of pairwise intensity distributions	Where higher values indicate level of disorder
	Maximal Correlation Coefficient (MCC)	Nonlinear dependency between intensities	Reflects texture complexity
	Maximum Probability (Joint maximum)	Dominance of the most common pixel pair	Indicates prevalence of specific local intensity patterns
	Sum Average	Mean of summed gray levels	Reflects global gray-level balance
	Sum Entropy	Randomness of summed intensity pairs	Captures overall disorder

	Sum Squares	Spread of intensity values	Corresponds to variability across pixel neighbors
Gray Level Run Length Matrix (GLRLM)	Gray Level Non-Uniformity	Variability of gray levels across runs	Where lower values indicate homogeneous intensity (i.e., smooth fibers)
	Gray Level Variance	Measure of variance in gray level intensity for runs	Variation of intensity values capturing level of fragmentation
	High Gray Level Run Emphasis	Distribution of higher intensity runs	Highlights dominance of bright structures
	Long Run Emphasis	Prevalence of longer runs	Captures elongated structures
	Long Run High Gray Level Emphasis	Long runs of high intensity	Reflects extended bright regions, corresponding to level of fragmentation
	Long Run Low Gray Level Emphasis	Long runs of low intensity	Reflects extended low intensity regions, corresponding to level of fragmentation
	Low Gray Level Run Emphasis	Distribution of lower intensity runs	Highlights dominance of low-intensity patterns
	Run Entropy	Randomness of run distributions	Where higher values correspond to heterogeneous textures
	Run Length Non-Uniformity	Variability in run lengths	Higher values correspond to irregular fiber lengths
	Run Percentage	Proportion of runs relative to total voxels	Quantifies coarseness
	Run Variance	Variability in run lengths	Captures level of fragmentation
	Short Run Emphasis	Prevalence of short, fragmented runs	Indicates fragmented structures
	Short Run High Gray Level Emphasis	Short runs of high intensity	Indicates fragmented bright structures
	Short Run Low Gray Level Emphasis	Short runs of low intensity	Reflects extended low intense regions
Gray Level Size Zone Matrix (GLSZM)	High Gray Level Zone Emphasis	Small zones of high intensity	Highlights fine and bright mitochondrial structures
	Large Area Emphasis	Frequency of large zones	Captures coarse structural organization

	Large Area High/Low Gray Level Emphasis	Joint distribution of large zones with high/low pixel intensities	Highlights extended homogeneous regions
	Low Gray Level Zone Emphasis	Small zones of low intensity	Reflects low intensity and fine-grained mitochondrial textures
	Size Zone Non-Uniformity	Variability in zone sizes	Where low values indicate greater uniformity
	Small Area Emphasis	Frequency of small zones	Reflects fine textures and granularity
	Small Area High/Low Gray Level Emphasis	Joint distribution of small zones with high/low pixel intensities	Captures fine texture complexity
	Zone Entropy	Randomness in zone distributions	Indicates textural disorder
	Zone Percentage	Ratio of zones to voxels	Where a higher value indicates finer mitochondrial textures
	Zone Variance	Variability of zone sizes	Captures structural heterogeneity
Neighbouring Gray Tone Difference Matrix (NGTDM)	Busyness	Rate of intensity change between neighbours	Where higher values reflect rapid local fluctuations in intensities
	Coarseness	Average intensity difference between a voxel and neighbours	Higher values indicate smoother textures
	Complexity	Number of primitive components or variation	Captures structural irregularity
	Contrast	Magnitude of intensity variation	Captures edges, where higher values mean stronger/brighter edges
	Strength	Degree of pronounced structures	Higher values correspond to bold coarse patterns
