# Supplementary material for: MitoTex (Mitochondria Texture Analysis User Interface): Open-Source Framework for Textural Characterization and Classification of Mitochondrial Structures
Source: Int J Mol Sci. 2026 Jan 24;27(3):1191. doi: 10.3390/ijms27031191 (PMC12897202; doi:10.3390/ijms27031191)
Supplement: Supplementary file 1 [file ijms-27-01191-s001.zip › Supplemental_material_S2.pdf]

Supplemental Material S2 - Machine Learning Feature Selection and Classification Workflow

1. Data Preparation and Feature Selection

To minimize the risk of overfitting and classification interpretability, the feature set of 93 features underwent data processing prior to classification:

1. **Data cleaning:** Missing values were imputed using the average of each feature.
2. **Standardization:** Features were normalized using a z-score transformation via *StandardScaler* to ensure comparable magnitudes.
3. **Feature selection using Recursive Feature Elimination (RFE):** RFE was applied as the feature selection strategy. RFE iteratively removes less informative features based on model performance until an optimal subset of features is retained.

This model-based approach ensures that only highly relevant and minimally redundant features were selected.

2. Recursive Feature Elimination (RFE)

RFE is a wrapper-based feature selection method that iteratively removes less important features while retraining the model, ultimately selecting the optimal subset

2.1 Implementation

- **Base estimator:** Random Forest Classifier (*n_estimators* = 100, *random_state*= 42)
- **Stopping criterion:** Iteratively eliminates features until the top 20 features are retained.
- **Step size:** Adjusted dynamically based on feature dimensionality (1 for $\leq$50 features, otherwise 10% of feature count).

4.2 Interpretation

RFE removes less important features, providing biologically interpretable results by ranking features by importance. For example, intensity-based features (mean, entropy, kurtosis) and texture features were frequently prioritized, highlighting structural differences in mitochondrial morphology.

3. Decision Tree (DT) Classifier

Decision Trees are interpretable models that split the features into subsets based on feature thresholds, forming a tree-like structure.

3.1 Implementation

The *DecisionTreeClassifier* from scikit-learn was used.

Hyperparameters tuned using GridSearchCV:

- **Criterion:** Gini impurity vs entropy
- **Max_depth:** None, 10, 20, 30, 40, 50
- **Min_samples_split:** 2, 5, 10
- **Min_samples_leaf:** 1, 2, 4

3.2 Training and Validation

The dataset was split into 70% training and 30% testing with stratification. Best hyperparameters were selected based on accuracy.

Model performance evaluated with:

- Repeated stratified 10-fold cross-validation across the full dataset, reporting mean ± standard deviation of accuracy
- Confusion matrices to assess misclassification
- ROC curves and AUC scores

3.3 Interpretation

Each split in the DT represents a decision rule derived from one feature, making this approach useful for biological interpretability. Such as, identifying intensity-based thresholds that separate mitochondria structures

4. Support Vector Machine (SVM) Classifier

SVMs are robust classifiers that maximize the margin between decision boundaries

4.1 Multi-class One versus Rest SVM (subset of 3 or more classes) Implementation

- One versus rest support vector machine (OvR-SVM) with a radial basis function (RBF) is selected, as mitochondrial texture features are not linearly separable
- Pipeline: Standardization → SVM; performance assessed using repeated stratified 2-fold CV × 3 repeats”

4.2 Binary SVM (subset of two classes) Implementation

- Binary SVM with RBF is selected
- Pipeline: Standardization → SVM; performance assessed using same repeated 2-fold CV

4.3 Training and Validation

- Dataset split: 67% training and 33% testing
- Repeated stratified k-fold cross-validation (2 splits x 3 repeats) for robust accuracy estimation

- Performance metrics included accuracy, F1-score, precision, recall, and ROC-AUC

4.4 Interpretation

SVM, while less interpretable than DT, often provides higher classification accuracy in complex and high dimensional data.
