# Supplementary material for: MitoTex (Mitochondria Texture Analysis User Interface): Open-Source Framework for Textural Characterization and Classification of Mitochondrial Structures
Source: Int J Mol Sci. 2026 Jan 24;27(3):1191. doi: 10.3390/ijms27031191 (PMC12897202; doi:10.3390/ijms27031191)
Supplement: Supplementary file 1 [file ijms-27-01191-s001.zip › Supplemental_material_S3.pdf]

Supplemental Material S3 - Machine Learning Classification Reports

Supplemental S3 Table S1- Classification report for multiclass DT of full feature set (mitochondrial structures). Cross-validation 0.79±0.03 and test accuracy of 0.76

	Precision	Recall	F1-Score	Support
Rods	0.73	0.77	0.75	64
Puncta	0.91	0.96	0.94	75
Fibers	0.66	0.55	0.60	42
Accuracy			0.80	181
Macro average	0.77	0.76	0.76	181
Weighted Average	0.79	0.80	0.79	181

Supplemental S3 Table S2- Classification report for multiclass SVM of full feature set (mitochondrial structures). Cross-validation 0.94±0.01 and test accuracy of 0.93

	Precision	Recall	F1-Score	Support
Rods	0.87	0.94	0.90	64
Puncta	1.00	0.96	0.98	75
Fibers	0.90	0.86	0.88	42
Accuracy			0.93	181
Macro average	0.92	0.92	0.92	181
Weighted Average	0.93	0.93	0.93	181

Supplemental S3 Table S3- Classification report for multiclass DT of RFE selected feature set (mitochondrial structures). Cross-validation 0.83±0.02 and test accuracy of 0.85

	Precision	Recall	F1-Score	Support
Rods	0.78	0.80	0.79	64
Puncta	0.97	0.95	0.96	75
Fibers	0.72	0.74	0.73	42
Accuracy			0.85	181
Macro average	0.83	0.83	0.83	181
Weighted Average	0.84	0.85	0.85	181

Supplemental S3 Table S4- Classification report for multiclass SVM of RFE selected feature set (mitochondrial structures). Cross-validation 0.91±0.02 and test accuracy of 0.85

	Precision	Recall	F1-Score	Support
Rods	0.84	0.92	0.88	64
Puncta	0.97	0.96	0.97	75
Fibers	0.92	0.81	0.86	42
Accuracy			0.91	181
Macro average	0.91	0.90	0.90	181
Weighted Average	0.91	0.91	0.91	181

Supplemental S3 Table S5- Classification report for binary DT of RFE selected feature set (mitochondrial structures). Cross-validation 0.83±0.04 and test accuracy of 0.78

	Precision	Recall	F1-Score	Support
Control	0.78	0.84	0.81	45
LPSIFN-γ	0.77	0.69	0.73	35
Accuracy			0.78	80
Macro average	0.77	0.77	0.77	80
Weighted Average	0.77	0.78	0.77	80

Supplemental S3 Table S6- Classification report for binary SVM of RFE selected feature set (mitochondrial structures). Cross-validation 0.86±0.02 and test accuracy of 0.88

	Precision	Recall	F1-Score	Support
Control	0.95	0.82	0.88	45
LPSIFN-γ	0.80	0.94	0.87	35
Accuracy			0.88	80
Macro average	0.88	0.88	0.87	80
Weighted Average	0.89	0.88	0.88	80
